# Lymphadenoma of the salivary gland: Report of 10 cases

**DOI:** 10.3892/ol.2014.1827

**Published:** 2014-01-24

**Authors:** GUANGLONG LIU, JIE HE, CHUNYE ZHANG, SHUITING FU, YUE HE

**Affiliations:** 1 Department of Oral and Maxillofacial-Head and Neck Oncology, Ninth People’s Hospital, Shanghai Jiao Tong University School of Medicine, Shanghai 200011, P.R. China; 2 Shanghai Key Laboratory of Stomatology, Ninth People’s Hospital, Shanghai Jiao Tong University School of Medicine, Shanghai 200011, P.R. China; 3 Department of Oral Pathology, Ninth People’s Hospital, Shanghai Jiao Tong University School of Medicine, Shanghai 200011, P.R. China

**Keywords:** lymphadenoma, non-sebaceous lymphadenoma, sebaceous lymphadenoma, diagnosis, pathology

## Abstract

Lymphadenoma of the salivary gland is rare, and the typical characteristics of lymphadenoma remain poorly understood. The aim of this study was to analyze the experience of a single institution in the clinical diagnosis, treatment and prognosis of this type of tumor of the salivary gland. All cases of lymphadenoma diagnosed at the institution between 1996 and 2012 were analyzed. The clinical information (including age, gender and tumor location, process of tumor development, imaging data, surgical treatment and follow-up information) and pathological features were evaluated. All tumors occurred in the parotid glands; three cases were sebaceous lymphadenoma (two male and one female) and seven were non-sebaceous lymphadenoma (three male and four female). The average ages of the patients were 68.3 and 42.4 years for the sebaceous and non-sebaceous groups, respectively. The majority of cases (90%) were diagnosed as pleomorphic adenoma or adenolymphoma prior to surgery, but were confirmed as lymphadenoma by pathological analysis following surgery. During the follow-up period, which ranged between 3 and 36 months with a mean of 30 months, no recurrence of the lesion was identified and the quality of life was good for each patient. In conclusion, the diagnosis of salivary gland lymphadenoma should be based on the clinical and, in particular, the pathological manifestations of the disease. Immunohistochemistry is considered as a practical and helpful adjuvant method of the diagnosis for this type of tumor. Complete surgical resection is the first choice of treatment. Further exploration of the histological origin of lymphadenoma of the salivary gland is necessary due to the insufficient number of reported cases.

## Introduction

Lymphadenoma, which is classified as sebaceous and non-sebaceous lymphadenoma based on the presence or absence of sebaceous differentiation, is a rare type of tumor of the salivary gland. In the majority of cases, this type of tumor presents as a painless mass of long duration, and the epithelial component comprises benign basal and squamous cells. Since the first report in 1960 by McGavran *et al* (1), to the best of our knowledge, <110 cases of the salivary gland have been reported in the English language literature. However, this may be due to diagnostic difficulty as this type of tumor partially resembles numerous other types of salivary gland neoplasm, including cystadenoma, Warthin’s tumor and pleomorphic adenoma; even mucoepidermoid carcinoma or metastatic adenocarcinoma may enter into the differential diagnosis (1–3). The present study reports a large series of cases of lymphadenoma of the salivary gland in the Chinese population, with a complete analysis of the clinical and pathological data to enable the discussion of the features of the clinical diagnosis and histogenesis in these cases.

## Patients and methods

### Clinical data

Ten consecutive patients with lymphadenoma in the parotid gland who were treated at the Department of Oral and Maxillofacial-Head and Neck Oncology, Ninth People’s Hospital, Shanghai Jiao Tong University School of Medicine (Shanghai, China) between 1996 and 2012 were retrospectively reviewed by their clinical data (including age, gender and tumor location, process of tumor development, imaging data and surgical treatment) and pathological features.

### Surgery

Following the signing of informed consent forms for the surgery, all patients received surgical resection of the masses with preservation of important neighboring structures, including the facial nerve, great auricular nerve, sternocleidomastoideus muscle, internal jugular vein and carotid arteries. All patients provided written informed consent for their participation in this study.

### Histological and immunohistochemical examination

A specimen from each patient was submitted for histological examination and, following fixation in formalin solution and inclusion in paraffin, 3–5-μm sections were stained with hematoxylin and eosin for conventional evaluation. The histopathological diagnoses of all patients following the surgery were lymphadenoma. Immunohistochemical examination was performed in all patients, including the detection of cytokeratin 8 (CK8), CK19, Ki-67, CKpan, S-100, smooth muscle actin and vimentin. All patients were followed up by a return visit with a follow-up period of 3–36 months. When the patients returned, routine physical examination was performed, and if any suspicious mass was present in the parotid gland and neck region, image examination was suggested. Fine needle aspiration biopsy was also suggested if necessary.

## Results

### Demographic data

As shown in Tables I and II, among the total 10 cases, five were male and five were female (ratio of the tumor sites, six left parotid gland to four right parotid gland). Three cases (two male and one female) were diagnosed with sebaceous lymphadenoma and seven (four female and three male) with non-sebaceous lymphadenoma. The ratio of the tumor sites was two left parotid gland to one right parotid gland for sebaceous lymphadenoma and four left parotid gland to three right parotid gland for non-sebaceous lymphadenoma. The mean age of all patients was 50.2 years, with a range of 10–75 years. Patients >50 years old accounted for 50% of the 10 patients and the ratio of sebaceous to non-sebaceous lymphadenoma in these cases was 3:2. Only one patient was a child; this was a 10-year-old male who was diagnosed with non-sebaceous lymphadenoma.

### Clinical study

**Figure 1:**
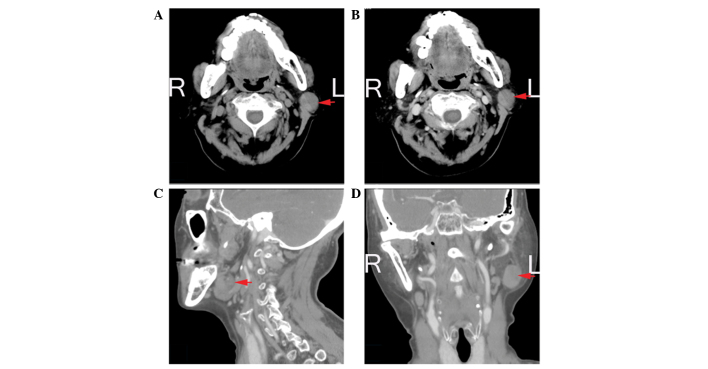
Computerized tomography scan of the non-sebaceous lymphadenoma of the fourth patient. The mass in the left parotid region was round in shape with a clear boundary and uniform in density, without bone destruction (red arrow). (A) Transverse and (B) enhanced scanning, and scans in the (C) sagittal and (D) coronal positions. R, right; L, left.

All tumors occurred in the parotid gland and presented as painless masses, which were slowly enlarging. The duration of the symptoms ranged from a few months to 20 years. Fig. 1 shows the non-sebaceous lymphadenoma computed tomography data of the fourth patient.

All patients underwent surgical therapy for the tumors. The parotid lesions were excised by superficial or complete parotidectomy with dissection and preservation of the facial nerve.

During the follow-up period, which ranged between 3 and 36 months with a mean of 30 months, no recurrence of the lesion occurred and the patient’s quality of life was good.

### Histological analysis

All tumors were observed to be well circumscribed and 8 of the 10 tumors (80%) were encapsulated. The contents in the cystic tumors were gelatinous and yellow sebum-like.

**Figure 2:**
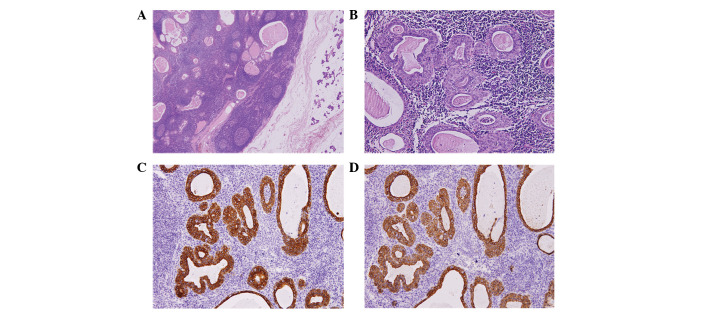
(A) Marked lymphoid stroma and epithelial component (staining, hematoxylin and eosin; magnification, ×40). (B) Two layers of cells, with no sebaceous differentiation and formation of lymphoid stroma follicles (staining, hematoxylin and eosin; magnification, ×200). Immunohistochemical analysis showed (C) CKpan+ and (D) CK8+ cells (magnification, ×200). CK, cytokeratin.

Microscopically, in the cases of sebaceous lymphadenoma the epithelial component comprised solid nests, tubular structures, glands, cords, and tubules of basal, glandular cells. There were always two cell layers, namely an outer basal cell layer and an inner luminal glandular cell layer that was typically composed of cuboidal or low columnar cells. This finding is similar to those of a previous study (2). In the cases of non-sebaceous lymphadenoma, significant lymphoid stroma and an epithelial component (Fig. 2A), which together formed solid islands, tubular structures and also lumens of different sizes, were visible. Similarly, two layers were detected in the cases of non-sebaceous lymphadenoma. The outer cells were flat, cubic or cylindrical. The chambers inside the outer layer, in which the formation of lymphoid stroma follicles was usually observed, contained eosinophilic red amorphous material, without sebaceous secretion (Fig. 2B).

Notably, no specific immunohistochemical indicators had been reported in previous studies, despite the salivary gland lymphadenoma having characteristic pathological features. However, in the present study, the majority of the tumors exhibited positive immunohistochemical staining of CK8 and CKpan, as shown in Fig. 2C and D.

## Discussion

Lymphadenoma, including sebaceous and non-sebaceous lymphadenoma, is a rare type of salivary gland tumor. In the present study, this type of tumor was observed to be well circumscribed and exhibited typically benign behaviors, with the majority of the cases affecting adults aged >30 years (80%). Of the 10 cases, approximately one-third exhibited sebaceous differentiation to an extent, which was the opposite to the findings of a previous study (3). The underlying specific reasons for this phenomenon are not yet clear.

Dardick and Thomas (4) previously described the following criteria for the diagnosis of non-sebaceous lymphadenoma: No sebaceous differentiation; no oncocytic epithelium; a predominant lymphocytic component; solid, glandular or cystic epithelial nests; and lack of nodal capsule or subcapsular sinusoids. Although lymphadenoma has been considered as a form of basal cell adenoma accompanied by a heavy lymphoid infiltrate (5), according to this perspective, lymphadenoma would be identified as a variant of other types of adenomatous epithelial tumors with a prominent lymphoid component, not a specific type of tumor. However, the majority of studies strongly support the view that lymphadenoma is an entity comprising a heterogeneous spectrum of epithelial differentiation and including a variable degree of cystic transformation (7,8,9). Numerous types of salivary gland tumor, including Warthin’s tumor, acinic cell carcinoma, acquired immunodeficiency syndrome-related lymphoepithelial cysts, lymphoepithelial carcinoma or mucoepidermoid carcinoma, often have large amounts of lymphoid stroma and epithelial neoplasms (6). Furthermore, the epithelium in non-sebaceous lymphadenoma is morphologically bland and does not infiltrate nearby tissue. Additionally, no mitotic activity and the lack of invasive tumor features support the diagnosis of non-sebaceous lymphadenoma (4).

The pathogenetic or histological origin of non-sebaceous lymphadenoma has been proposed to be embryonic salivary gland inclusions in the intraparotid or periparotid lymph nodes. A strong argument for this hypothesis are that studies have identified an unequivocal hilus structure with embryonic parenchymal inclusions, in conjunction with frequent secondary follicles and lymph vessels within the marginal sinus structures (2,3,7,10). However, this theory is in conflict with the majority of studies, which instead regard the lymphoid component as reactive tumor-associated lymphoid tissue (6,8,11–13). According to the present study, only lymphoid component accompanied with epithelial tissue was identified, which provides positive evidence for the aforementioned second view. However, this is uncertain due to the insufficient number of samples in the present study.

Thus far, to the best of our knowledge, only 37 cases of non-sebaceous lymphadenoma have been reported in the English language literature (2,4,6–8,10–16); thus, the seven cases described in the present study increases the total number of reported cases to 44. Although the present study shared numerous equivalent findings with those of the previous studies, there are also noteworthy differences. For example, one case in the present study was in a 10-year-old male, which, to the best of our knowledge, is the youngest case of non-sebaceous lymphadenoma to be reported (9,10,16–18). Lymphadenoma may be rare in children since sebaceous differentiation in the salivary glands develops later in life and is not present in infants and children (19).

Overall, the present study reported 10 cases of lymphadenoma, in which the patients typically presented with a painless mass for which complete surgical excision appeared to be curative, and attempted to discuss an exact method of diagnosis for this type of tumor, particularly for non-sebaceous lymphadenoma. Due to the similarity of the clinical features of lymphadenoma with those of other types of parotid tumor, cases of lymphadenoma are often misdiagnosed and there are no remarkable clinical features that completely distinguish lymphadenoma from other types of parotid tumor. The most effective approach of identification is by the pathology of the tumor. However, the exact mechanisms of the tumorigenesis of non-sebaceous lymphadenoma from salivary gland inclusions remain obscure. As there are few studies concerning cases of lymphadenoma, there is a lack of knowledge of this type of tumor. An improved understanding and a more in-depth investigation of lymphadenoma in the salivary gland requires further studies including larger numbers of cases.

## Figures and Tables

**Table I:** Sebaceous and non-sebaceous lymphadenomas: Clinical information.

Case	Age (years)	Gender	Site	Presentation	Size (cm)	Treatment	Pre-surgery diagnosis	Follow-up (months)	Recurrence
S 1	73	M	L parotid gland	Mass for 6 months	2.3×1.8	Surgical resection	Pleomorphic adenoma	24	No
S 2	60	M	R parotid gland	Mass for 3 months	4.0×3.0	Surgical resection	Pleomorphic adenoma	36	No
S 3	72	F	L parotid gland	Mass for 20 years	3.0×2.0	Surgical resection	Lymphadenoma	36	No
NS 1	20	M	R parotid gland	Mass for 6 years	2.0×3.0	Surgical resection	Pleomorphic adenoma	36	No
NS 2	39	F	R parotid gland	Mass for 5 years	2.0×2.0	Surgical resection	Pleomorphic adenoma	36	No
NS 3	75	F	L parotid gland	Mass for 20 years	5.0×6.0	Surgical resection	Pleomorphic adenoma	28	No
NS 4	70	F	L parotid gland	Unknown	2.5×2.0	Surgical resection	Pleomorphic adenoma	36	No
NS 5	33	F	L parotid gland	Mass for 3 years	2.0×1.5	Surgical resection	Pleomorphic adenoma	36	No
NS 6	48	M	L parotid gland	Mass for 1 month	2.0×3.0	Surgical resection	Pleomorphic adenoma	36	No
NS 7	10	M	R parotid gland	Mass for 8 months	1.0×1.0	Surgical resection	Pleomorphic adenoma	36	No

Surgical resection means that the parotid lesions were excised by superficial or profound parotidectomy with facial nerve dissection and preservation. S, sebaceous lymphadenoma; NS, non-sebaceous lymphadenoma; F, female; M, male; L, left; R, right.

**Table II:** All lymphadenoma cases.

Lymphadenoma type	Mean age (range; years)	Gender ratio
Sebaceous (n=3)	68.3 (60–73)	1 F:2 M
Non-sebaceous (n=7)	42.4 (10–75)	4 F:3 M
All (n=10)	50.2 (10–75)	5 F:5 M

F, female; M, male.
